# Infection Related Never Events in Pediatric Patients Undergoing Spinal Fusion Procedures in United States: Prevalence and Predictors

**DOI:** 10.1371/journal.pone.0077540

**Published:** 2013-11-05

**Authors:** Veerajalandhar Allareddy, Veerasathpurush Allareddy, Romesh P. Nalliah, Sankeerth Rampa, Min Kyeong Lee

**Affiliations:** 1 Department of Pediatric Critical Care, Case Western Reserve University, Cleveland, Ohio, United States of America; 2 Department of Orthodontics, College of Dentistry – The University of Iowa, Iowa City, Iowa, United States of America; 3 Restorative Dentistry and Biomaterials Sciences, Harvard School of Dental Medicine, Boston, Massachusetts, United States of America; 4 Texas A & M University, College Station, Texas, United States of America; 5 Harvard School of Dental Medicine, Boston, Massachusetts, United States of America; Johns Hopkins Bloomberg School of Public Health, United States of America

## Abstract

**Objective:**

To examine the prevalence and predictors of infection related never events (NE) associated with spinal fusion procedures (SFP) in children (age < = 18 years) in the United States.

**Methods:**

We performed a retrospective analysis of the Nationwide Inpatient Sample for the years 2004 to 2008. All pediatric hospitalizations that underwent SFP were selected for analysis. The main outcomes measures include occurrence of certain NE’s. The association between the occurrence of a NE and factors (patient & hospital related) were examined using multivariable logistic regression analysis.

**Results:**

Of 56,465 hospitalizations, 61.7% occurred among females. The average age was 13.7 y and two-thirds were whites. The major insurance payer was private insurance (67.4%). About 82% of all hospitalizations occurred on an elective basis. Teaching hospitals accounted for a majority of hospitalizations (87.9%). Two-thirds were posterior fusion techniques, 52.3% had underlying musculoskeletal deformities, and the most frequently present co-morbid conditions (CMC) included paralysis (10.9%), chronic pulmonary disease (9.7%), and fluid/electrolyte disorders (7.6%). Overall rate of occurrence of a NE was 4.8%. Post-operative pneumonia was the most frequently occurring NE (2.9%). Female gender (OR = 0.78) and elective admissions (OR = 0.66) were associated with lower risk of NE occurrence. Medicaid coverage (OR = 1.46), primary diagnosis of other acquired deformities (OR = 1.82), spinal cord injury (OR = 6.94), other nervous system disorders (OR = 2.84) were associated with higher risk of NE occurrence. Among CMC, those with chronic blood loss anemia (OR = 2.57), coagulopathy (OR = 1.97), depression (OR = 2), drug abuse (OR = 3.71), fluid/electrolyte disorders (OR = 2.62), neurological disorders (OR = 1.72), paralysis (OR = 1.75), renal failure (OR = 5.45), and weight loss (OR = 4.61) were risk factors for higher odds of a NE occurrence. Hospital teaching status, region, hospital size, and patient race did not influence the occurrence of NE.

**Conclusion:**

The never events examined in the current study occurred in 4.8% of children hospitalized with SFP. Certain predictors of NE are identified in this study.

## Introduction

Spinal fusions are performed to alleviate pain, correct deformities, reduce neurological deficits or treat acute problems like fracture. A 2012 epidemiological study of the hospitalizations due to spinal fusions showed that there was an increase in prevalence from 1998 to 2008 in the United States [1]. Additionally, the frequency and hospital charges associated with these hospitalizations increased at a rate higher than several other inpatient procedures including hip replacement, knee arthroplasty, and coronary angioplasty.

Complications associated with spinal fusion have been considered in a limited number of studies. Trends in anterior cervical decompression and spinal fusion were studied over the 1990–2004 time period [2]. There was an eight fold increase in prevalence and a reduction in mean length of stay from 5.17 days to 2.38 days. Additionally, complication rate declined from 4.6% to 3.03%. Another study examined patients undergoing spinal surgery and found that 3.04% suffered wound infections and postoperative infection was associated with considerably longer hospital stays (7.12 days) compared to absence of postoperative infection (4.20 days) [3]. A case-control study compared 13 patients with surgical site infection to 47 without the infection after laminectomy, spinal fusion surgery, or both [4]. The median hospital stay was longer in case-patients (16 days compared to 4 days), with $12,477 excess cost per case and 11 excess days in length of stay. A multi-center study of cervical spine surgeries demonstrated that there was no linear trend over the five years of the study and that complication rate was approximately 5% [5].

A medical complication is an unfavorable event during medical care of a patient, whereas the term never event was coined by Ken Kizer [6], the former CEO of the National Quality Forum, to describe a medical outcome that should never occur. While some of the never events, such as wrong site surgery, accidentally leaving foreign bodies during surgery, and post-operative falls and hip fractures, may be attributed to deficiencies in process of care, there are other designated never events (such as post-operative pneumonia, *Clostridium difficile* infection, infection with microorganisms resistant to penicillin, post-operative infections, and decubitus ulcers) which could occur due to the immuno-compromised nature of the patient or any other patient level attribute rather than an obvious deficiency in the care process at the hospital [7]. To our knowledge there is no published research on occurrence of the above mentioned infection-related never events in patients undergoing spinal fusion using a nationwide dataset. The objective of this study is to examine prevalence and predictors of infection-related never events associated with spinal fusions in pediatric population (≤18 years of age) in the United States.

## Materials and Methods

### Description of Nationwide Inpatient Sample

We used discharge data from the Nationwide Inpatient Sample (NIS) dataset, a component of the Healthcare Cost and Utilization Project (HCUP) provided by the Agency for Healthcare Research and Quality (AHRQ) [8]. The NIS, a sample of all nonfederal acute-care general hospitals in the United States, is the largest all-payer inpatient care database in the United States [8]. The NIS dataset has information on a multitude of patient and hospital level variables including age, gender, race, reason for hospitalization, secondary diagnoses, procedures performed during hospitalization, length of stay in hospital, hospital charges, type of admission, insurance status, and hospital level variables (teaching status, bed size, and geographic location) A retrospective analyses on the NIS datasets from the years 2004 to 2008 was performed.

As per University Hospitals Case Medical Center institutional review board (IRB) and in agreement with Federal Regulations 45 CFR 46.101 (b) which states “research involving the collection or study of existing data, documents, records, pathological specimens, or diagnostic specimens, if these sources are publicly available or if the information is recorded by the investigator in such a manner that subjects cannot be identified, directly or through identifiers linked to the subjects,” such studies are permitted to be classified as research that is “exempt” from IRB full or expedited review. IRB was not consulted for approval since the current study was a retrospective analysis of hospital based discharge dataset that is available publicly for purchase from Agency for Healthcare Research and Quality (AHRQ). The first author (VA) completed the data user agreement with HCUP-AHRQ and the obtained data. The HCUP-AHRQ data user agreement precludes reporting individual cell counts ≤10 to preserve patient confidentiality. Consequently, these numbers were not reported in the current study.

### Data User Agreement

The first author (VJA) signed and submitted the Data User Agreement with AHRQ prior to obtaining access to the NIS data. In accordance with the Data User Agreement, any given cell of tabulated data less than or equal to 10 were not reported to preserve patient confidentiality and were denoted by “DS,” which refers to discharge information suppressed.

### Selection of Cases for Analysis

All hospitalizations (≤18 years of age) that underwent a spinal fusion procedure were selected for analysis. International Classification of Disease, 9^th^ edition, clinical modification (ICD-9-CM) procedure codes were used to select the spinal fusion procedures. The types of spinal fusion procedures examined included the following –spinal fusion: not otherwise specified (ICD-9-CM procedure code 81.00), atlas-axis spinal fusion (81.01), other cervical fusion: anterior technique (81.02), other cervical fusion: posterior technique (81.03), dorsal and dorso-lumbar fusion: anterior technique (81.04), dorsal and dorso-lumbar fusion: posterior technique (81.05), lumbar and lumbo-sacral fusion: anterior technique (81.06), lumbar and lumbo-sacral fusion: lateral transverse process technique (81.07), lumbar and lumbo-sacral fusion: posterior technique (81.08), re-fusion of spine: not otherwise specified (81.30), re-fusion of atlas-axis spine (81.31), re-fusion of other cervical spine: anterior technique (81.32), re-fusion of other cervical spine: posterior technique (81.33), re-fusion of dorsal and dorso-lumbar spine: anterior technique (81.34), re-fusion of dorsal and dorso-lumbar spine: posterior technique (81.35), re-fusion of lumbar and lumbo-sacral spine: anterior technique (81.36), re-fusion of lumbar and lumbo-sacral spine: lateral transverse process technique (81.37), re-fusion of lumbar and lumbo-sacral spine: posterior technique (81.38), and re-fusion of spine: not elsewhere classified (81.39).

### Data Elements Examined

#### Outcome variable

The outcome variable of interest in the current study was the occurrence of any of the following infection-related never events: post-operative pneumonia (ICD-9-CM diagnosis codes 481, 482, 485, 486, 507), decubitus ulcers (707), infection with microorganisms resistant to penicillin (V09.0), *Clostridium difficile* infection (008.45), post-operative infections (998.59), infection and inflammatory reaction due to vascular device, implant, or graft (996.62), and infection and inflammatory reaction due to indwelling urinary catheter (999.64). The occurrence of these never events was identified by using the ICD-9-CM diagnosis codes in the secondary diagnoses fields (n = 14 fields) and was coded as a binomial variable (yes or no). The ICD-9-CM codes to identify the never events were based on previously published findings [7].

#### Independent variables

The independent variables of interest included a set of factors at both patient level (age, gender, type of admission, insurance status, race/ethnicity, type of spinal fusion performed, type of primary diagnoses, and presence of co-morbid conditions) and hospital level (hospital teaching status, bed size, and hospital location). Insurance status included Medicare, Medicaid, private insurance, uninsured, and other insurance plans, including workers compensation, CHAMPUS, CHAMPVA, Title V, and other government programs. Primary diagnoses selected by Clinical Classification Software (CCS) codes were also examined: bone disease and musculoskeletal deformities (CCS code 212), other acquired deformities (209), other congenital anomalies (217), other fractures (231), spinal cord injury (227), spondylosis; intervertebral disc disorders; other back problems (205), complication of device; implant or graft (237), and other nervous system disorders (95). Co morbid conditions were obtained using the NIS disease severity measurement files.

### Analytical Approach

Characteristics of hospitalizations undergoing spinal fusion procedures were summarized using descriptive statistics. Odds of a hospitalization having at least one of the never events was analyzed by a multivariable logistic regression model using the Taylor linearization method “with replacement design” to compute the variances. Each hospitalization was used as the unit of analysis, and the NIS hospital stratum as the stratification unit. Clustering effects of outcomes within hospitals were adjusted in all analyses. A p-value of <0.05 was set *a priori* to be statistically significant. All statistical analyses were two-sided and were performed using SAS version 9.3 software (SAS Institute, Cary, North Carolina) and SAS Callable SUDAAN version 10.0.1 software.

## Results

A total of 56,465 hospitalizations that underwent a spinal fusion procedure occurred among children aged ≤18 years during the study period (years 2004 through 2008). Of these 61.7% occurred among females [Table 1]. The average age at the time of hospitalization was 13.7 years. Majority of the hospitalizations occurred on an elective basis (82.1%). Private insurance plans (67.4% of all hospitalizations) were the major primary payer followed by Medicaid (25.2%), other insurance plans (5.3%), and Medicare (0.2%). Uninsured comprised 1.9% of hospitalizations. Among those for whom information regarding race was available, whites comprised 66.8% of hospitalizations while blacks (12.6%), Hispanics (12.4%), Asians/Pacific islanders (2.4%), Native Americans (0.8%), and Other races (5%) accounted for the rest. Majority of hospitalizations (87.9%) occurred in teaching hospitals and large bed hospitals (58.7%). Hospitals located in southern regions of the United States accounted for 35.6% of hospitalizations.

**Table 1:** Characteristics of Hospitalizations Undergoing Spinal Fusion Procedures.

Characteristic	Frequency (%) [Total N = 56465]
**Gender**	
Male	21560 (38.3%)
Female	34746 (61.7%)
Missing	159
**Type of Admission**	
Emergency/Urgent	10101 (17.9%)
Elective	46199 (82.1%)
Missing	165
**Insurance**	
Medicare	107 (0.2%)
Medicaid	14183 (25.2%)
Private Insurance	37960 (67.4%)
Uninsured	1089 (1.9%)
Other insurance plans	3007 (5.3%)
Missing	119
**Race**	
White	25278 (66.8%)
Black	4753 (12.6%)
Hispanic	4689 (12.4%)
Asian/Pacific Islander	903 (2.4%)
Native American	307 (0.8%)
Other Races	1904 (5%)
Missing Information	18630
**Hospital Teaching Status**	
Non-teaching	6848 (12.1%)
Teaching	49617 (87.9%)
**Hospital Bed Size**	
Small	10289 (18.2%)
Medium	13039 (23.1%)
Large	33138 (58.7%)
**Hospital Region**	
Northeast	12099 (21.4%)
Midwest	13580 (24%)
South	20117 (35.6%)
West	10668 (18.9%)
**Mean Age In Years**	13.7

The percentages may not add up to 100% because of rounding to nearest single digit decimal place.

The different spinal fusion procedures performed during hospitalization are summarized in Table 2. The three most frequently performed procedure was dorsal and dorso-lumbar fusion – posterior technique (67.5% of hospitalizations), followed by lumbar and lumbo-sacral fusion- posterior technique (16.6%), and dorsal and dorso-lumbar fusion – anterior technique (11.2%).

**Table 2:** Types of Spinal Fusions Performed During Hospitalization.

Type of Spinal Fusion (ICD-9-CM Procedure Code)	Frequency (%)
Spinal fusion, not otherwise specified (81.00)	443 (0.8%)
Atlas-axis spinal fusion (81.01)	1343 (2.4%)
Other cervical fusion, anterior technique (81.02)	2681 (4.7%)
Other cervical fusion, posterior technique (81.03)	2709 (4.8%)
Dorsal and dorsolumbar fusion, anterior technique (81.04)	6338(11.2%)
Dorsal and dorsolumbar fusion, posterior technique (81.05)	38099 (67.5%)
Lumbar and lumbosacral fusion, anterior technique (81.06)	1582 (2.8%)
Lumbar and lumbosacral fusion, lateral transverse process technique (81.07)	313 (0.5%)
Lumbar and lumbosacral fusion, posterior technique (81.08)	9395(16.6%)
Refusion of spine, not otherwise specified (81.30)	34 (0.06%)
Refusion of atlas-axis spine (81.31)	69 (0.1%)
Refusion of other cervical spine, anterior technique (81.32)	34 (0.06%)
Refusion of other cervical spine, posterior technique (81.33)	114 (0.2%)
Refusion of dorsal and dorsolumbar spine, anterior technique (81.34)	142 (0.2%)
Refusion of dorsal and dorsolumbar spine, posterior technique (81.35)	1150 (2%)
Refusion of lumbar and lumbosacral spine, anterior technique (81.36)	88 (0.1%)
Refusion of lumbar and lumbosacral spine, lateral transverse process technique (81.37)	18 (0.03%)
Refusion of lumbar and lumbosacral spine, posterior technique (81.38)	618 (1.1%)
Refusion of spine, not elsewhere classified (81.39)	81 (0.1%)

The total numbers will not add to 56465 since any hospitalization might have underwent multiple spinal fusion procedures.

Among all the different diagnostic conditions, eight major conditions grouped by the Clinical Classification Software Codes (combines families of ICD-9-CM codes into groups based on groups of medical conditions) that were listed as the primary diagnosis for hospitalizations included other bone disease and musculoskeletal deformities (52.3% of hospitalizations), other acquired deformities (13.4%), other congenital anomalies (10.4%), other fractures (6.3%), spinal cord injury (4.7%), spondylosis including intervertebral disc disorders and other back problems (2.7%), complications of devices including implants and grafts (2.5%), and other nervous system disorders (1.8%) [Table 3].

**Table 3:** Frequently Reported Primary Diagnosis.

Primary Diagnosis (Clinical Classification Software Code)	Frequency (%)
Bone disease and musculoskeletal deformities (212)	29510 (52.3%)
Other acquired deformities (209)	7567 (13.4%)
Other congenital anomalies (217)	5888 (10.4%)
Other fractures (231)	3549 (6.3%)
Spinal cord injury (227)	2638 (4.7%)
Spondylosis; intervertebral disc disorders; other back problems (205)	1518 (2.7%)
Complication of device; implant or graft (237)	1437 (2.5%)
Other nervous system disorders (95)	1044 (1.8%)
All other conditions	3314 (5.9%)

Presence of the different co-morbid conditions based on the disease severity files available in the NIS datasets were examined in patients undergoing the spinal fusion procedures and their prevalence estimates are provided in Table 4. The five most frequently present co-morbid conditions included paralysis (10.9% of hospitalizations), chronic pulmonary disease (9.7%), fluid and electrolyte disorders (7.6%), neurological disorders (7.4%), and deficiency anemia’s (7.1%).

**Table 4:** Presence of Co-Morbid Conditions.

Co-morbid Condition	All Hospitals
AIDS	DS
Alcohol abuse	261(0.5%)
Deficiency anemia’s	4025 (7.1%)
Rheumatoid arthritis/collagen vascular diseases	122 (0.2%)
Chronic blood loss anemia	444(0.8%)
Congestive heart failure	51 (0.1%)
Chronic pulmonary disease	5500 (9.7%)
Coagulopathy	1504 (2.7%)
Depression	867 (1.5%)
Diabetes, uncomplicated	184 (0.3%)
Diabetes with chronic complications	DS
Drug abuse	349 (0.6%)
Hypertension	790 (1.4%)
Hypothyroidism	387 (0.7%)
Liver disease	40 (0.07%)
Lymphoma	DS
Fluid and electrolyte disorders	4286 (7.6%)
Metastatic cancer	60 (0.1%)
Neurological disorders	4201 (7.4%)
Obesity	939 (1.7%)
Paralysis	6165 (10.9%)
Peripheral vascular disorders	111 (0.2%)
Psychoses	413 (0.7%)
Pulmonary circulation disorders	62 (0.1%)
Renal failure	78 (0.1%)
Solid tumor without metastasis	127 (0.2%)
Peptic ulcer disease excluding bleeding	DS
Valvular disease	810 (1.4%)
Weight loss	405 (0.7%)

DS: discharge information suppressed as per data user agreement with AHRQ.

Occurrence of different infectious related never events were examined from the secondary diagnoses fields in the NIS datasets. The prevalence estimates of these events are presented in Table 5. Post-operative pneumonia (2.9% of all hospitalizations) was the most frequently prevalent infection related never event, followed by decubitus ulcers (1%), other post-operative infections (0.8%), Clostridium difficile infection (0.3%), infection and inflammatory reactions due to vascular device, implants, or graft (0.3%), infection with microorganisms resistant to penicillin (0.2%), and infection and inflammatory reaction due to indwelling urinary catheter (0.04%). One of these never events occurred in 4.8% of hospitalizations.

**Table 5:** Prevalence of Never Events.

Never Event (ICD-9-CM Diagnosis Code)	All Hospitalizations
Post-operative Pneumonia (481, 482, 485, 486, 507)	1625 (2.9%)
Decubitus Ulcers (707)	555 (1%)
Post-operative infections (998.59)	475 (0.8%)
Infection with microorganisms resistant to penicillin (V09.0)	120 (0.2%)
*Clostridium difficile* infection (008.45)	171 (0.3%)
Infection and inflammatory reaction due to vascular device, implant, or graft (996.62)	159 (0.3%)
Infection and inflammatory reaction due to indwelling urinary catheter (999.64)	25 (0.04%)
Any One of the Above Event	2720 (4.8%)

DS: discharge information suppressed as per data user agreement with AHRQ.

Results of the multivariable logistic regression analysis examine the simultaneous association between multiple patient and hospital level characteristics are summarized in Table 6. Females were associated with a lower odds of at least one never event compared to males (OR = 0.78, 95% CI = 0.65–0.94, p = 0.008). Each 1 year increase in age was associated with a lower odds for a never event (OR = 0.97, 95% CI = 0.94–1.00, p = 0.02). Elective admissions were associated with a lower odds for a never event compared to admissions occurring on emergency/urgent basis (OR = 0.66, 95% CI = 0.46–0.95, p = 0.02). Hospitalizations with Medicaid coverage were associated with a higher odds for a never event compared to those covered by private insurance plans (OR = 1.46, 95% CI = 1.17–1.83, p = 0.0008). Hospitalizations with a primary diagnosis of other acquired deformities (OR = 1.82, 95% CI = 1.24–2.67, p = 0.002), spinal cord injury (OR = 6.94, 95% CI = 4.37–11.01, p<0.0001), other nervous system disorders (OR = 2.84, 95% CI = 1.68–4.79, p = 0.0001), and all other conditions (OR =  = 4.22, 95% CI = 3.06–5.84, p<0.0001) were associated with higher odds for a never event compared to other bone disease and musculoskeletal deformities. Hospitalizations with chronic blood loss anemia (OR = 2.57, 95% CI = 1.17–5.66, p = 0.002), coagulopathy (OR = 1.97, 95% CI = 1.31–2.97, p = 0.001), depression (OR = 2.00, 95% CI = 1.14–3.52, p = 0.01), drug abuse (OR = 3.71, 95% CI = 1.72–8.00, p = 0.001), fluid and electrolyte disorders (OR = 2.62, 95% CI = 2.04–3.37, p<0.0001), neurological disorders (OR = 1.72, 95% CI = 1.23–2.41, p = 0.002), paralysis (OR = 1.75, 95% CI = 1.32–2.33, p = 0.0001), renal failure (OR = 5.45, 95% CI = 1.57–18.84, p = 0.007), and weight loss (OR = 4.61, 95% CI = 2.48–8.57, p<0.0001) were associated with a significantly higher odds for having a never event when compared to those without the respective co-morbid conditions.

**Table 6:** Predictors of Never Events.

Characteristic	Odds Ratio (95% CI)	p-value
**Age –1 year increase**	0.97 (0.94–1.00)	0.02
**Gender**		
Female	0.78 (0.65–0.94)	0.008
Male	Reference	
**Type of admission**		
Elective	0.66 (0.46–0.95)	0.02
Emergency/Urgent	Reference	
**Insurance**		
Medicare	-	-
Medicaid	1.46 (1.17–1.83)	0.0008
Other insurance	0.77 (0.46–1.29)	0.32
Uninsured	0.96 (0.52–1.78)	0.91
Private insurance	Reference	
**Race**		
Black	1.00 (0.71–1.40)	0.98
Hispanic	1.27 (0.89–1.80)	0.19
Asian/Pacific Islander	0.90 (0.35–2.26)	0.82
Native American	0.71 (0.30–1.68)	0.43
Other Races	0.77 (0.35–1.71)	0.52
Missing Information	0.85 (0.66–1.10)	0.22
White	Reference	
**Primary diagnosis**		
Other acquired deformities	1.82 (1.24–2.67)	0.002
Other congenital anomalies	1.17 (0.76–1.81)	0.46
Other fractures	1.25 (0.72–2.17)	0.42
Spinal cord injury	6.94 (4.37–11.01)	<0.0001
Spondylosis; intervertebral disc disorders; other back problems	0.92 (0.39–2.20)	0.85
Complication of device; implant or graft	1.51 (0.76–3.03)	0.24
Other nervous system disorders	2.84 (1.68–4.79)	0.0001
Other conditions	4.22 (3.06–5.84)	<0.0001
Other bone disease and musculoskeletal deformities	Reference	
**Co-morbid conditions**		
Alcohol abuse	0.80 (0.31–2.07)	0.65
Deficiency anemia’s	1.21 (0.85–1.74)	0.29
Rheumatoid arthritis/collagen vascular diseases	0.58 (0.07–4.84)	0.62
Chronic blood loss anemia	2.57 (1.17–5.66)	0.02
Congestive heart failure	1.48 (0.18–12.42)	0.72
Chronic pulmonary disease	1.16 (0.87–1.56)	0.31
Coagulopathy	1.97 (1.31–2.97)	0.001
Depression	2.00 (1.14–3.52)	0.01
Diabetes, uncomplicated	2.65 (0.79–8.87)	0.11
Drug abuse	3.71 (1.72–8.00)	0.001
Hypertension	1.34 (0.75–2.41)	0.32
Hypothyroidism	1.67 (0.66–4.20)	0.28
Liver disease	5.00 (0.88–28.34)	0.07
Fluid and electrolyte disorders	2.62 (2.04–3.37)	<0.0001
Metastatic cancer	0.00	
Neurological disorders	1.72 (1.23–2.41)	0.002
Obesity	0.63 (0.28–1.39)	0.25
Paralysis	1.75 (1.32–2.33)	0.0001
Peripheral vascular disorders	1.50 (0.28–8.22)	0.64
Psychoses	1.42 (0.71–2.83)	0.32
Pulmonary circulation disorders	4.03 (0.87–18.59)	0.07
Renal failure	5.45 (1.57–18.84)	0.007
Solid tumor without metastasis	0.72 (0.09–5.88)	0.76
Valvular disease	1.14 (0.53–2.44)	0.73
Weight loss	4.61 (2.48–8.57)	<0.0001
**Hospital region**		
Northeast	1.17 (0.86–1.57)	0.32
Midwest	0.88 (0.65–1.20)	0.42
South	1.04 (0.79–1.38)	0.76
West	Reference	
**Hospital teaching status**		
Teaching	1.13 (0.83–1.54)	0.43
Non-teaching	Reference	
**Hospital bed size**		
Large bed	1.17 (0.93–1.46)	0.17
Small/Medium bed	Reference	
**Year**		
2004	1.21 (0.87–1.70)	0.26
2005	1.58 (1.15–2.18)	0.005
2006	1.41 (1.00–1.99)	0.047
2007	0.90 (0.65–1.24)	0.51
2008	Reference	

## Discussion

The current study revealed certain characteristics in pediatric patients undergoing spinal fusion procedures are important predictors of the occurrence of six analyzed infectious and one non-infectious (decubitus ulcer) never events. The findings in this study are consistent with prior published study in an adult population who underwent major surgical procedures [7]. To our knowledge this is the first study to provide national estimates of such events in pediatric population undergoing a surgical procedure for spinal fusion.

In accordance with a prior study, the posterior approach to dorsal and dorso-lumbar fusion in children accounted for two thirds of the cases of spinal fusion procedures [9]. In a series of 191 patients who were treated with posterior instrumentation and fusion in children, the overall incidence of deep wound infection was 6.2% [9]. Similarly, in a large series of pediatric patients who underwent posterior spinal fusion, the mean annual risk of surgical site infection was comparable, at 4.4% [10]. Risk factors associated with increased risk included as ASA score greater than 2, obesity (body mass index [BMI] greater than 95^th^ percentile, antibiotic prophylaxis with clindamycin). Hypothermia during the surgery appeared to provide protection against surgical site infection in this population [10]. Other studies reported the risk of surgical site mean annual infection rate as ranging from 1.3 to 6.3%[11]–[14]. The low overall prevalence of any of the never events found in this study could be attributed to the elective nature of the procedure which accounted for the majority of the cases (82%), optimal post-operative care, as well as favorable patient or hospital characteristics. Another factor that could be contributing to the low rates is under-reporting of never events. However, this needs further empirical support. The lower risk (OR = 0.66) of developing never events exhibited in patients undergoing elective procedures may be attributed to careful pre-operative risk assessment, including co-morbidity identification and potential risk modification to reduce or eliminate infectious events.

In the current study, patients who had a primary diagnosis of spinal cord injury had seven times higher risk of developing a never event. Spinal cord injury may necessitate prolonged periods of immobilization, mechanical ventilator support, and higher risk of complications such as paralysis. Immobilization is a known independent risk factor for decubitus ulcers formation. In their retrospective study of pediatric patients, Jain et al. examined outcomes in patients undergoing posterior spinal fusion surgery and demonstrated that patient diagnosis predicts blood loss [15]. A large retrospective study showed that in children with nervous system disorders (such as cerebral palsy and scoliosis), the unit rod instrumentation technique to correct scoliosis was associated with lower complications [16]. Patients in this current study with nervous system disorders had a significantly higher odds (OR = 2.84) of developing a never event. Since the information on the type of instrumentation is not available in the NIS database, it is impossible to assess the role of a particular type or technique in preventing or minimizing never events in a particular group of patients, such as those with nervous system disorders.

Patients who simultaneously had weight loss were at a significantly higher risk (4.6 times) of developing a never event. Weight loss and associated adverse co-morbidities could be related to poor nutritional status, which in turn may lead to decreased or ineffective immunity leading to increased risk of infections. Prior studies have demonstrated that the weight loss is associated with higher mortality and higher risk for complications after coronary artery bypass graft surgery [17], [18]. In contrast, obesity (BMI>95%) was associated with a higher risk of surgical site of infection in a series of patients who underwent posterior spinal fusion [10]. In prior studies, obesity was associated with an increased risk of hospital acquired infections among surgical patients [19], [20]. However, the results of this study showed no association between obesity and never events.

Depression was associated with two-fold higher odds of a never event. Evidence exists that various immunological factors are compromised in chronic stress and depression that predispose to increased risk of infection [21]–[23]. Patients who were paralyzed had higher odds of never event (1.75) and could represent a group of patients where risk modification may be difficult to attain but may represent a population where other modifiable risks optimization is of paramount importance before elective procedures. However, this awaits further empirical support.

There are several limitations to this study pertaining to the use of a large secondary hospital discharge administrative datasets, retrospective study design, and case selection. Prior study has shown efficient use of administrative data combining with clinical data to stratify surgical risk [24], [25]. While the study used multivariable logistic regression analysis to account for the confounding effects of patient- and hospital-level variables, the risk adjustment performed is not comprehensive owing to the lack of adequate clinical data (e.g. PRISM scores) in the NIS dataset. There may be coding inaccuracies and inconsistencies within and across hospitals. The current study is a retrospective analysis of hospital discharge datasets and therefore the cause and effect relationship between the independent variables and occurrence of a never event cannot be established. Finally, the selection of cases was based on the procedures performed during hospitalization, and consequently the sample is heterogeneous with multiple reasons for hospitalization and severity of disease.

This study explores the potential predictors of occurrence of seven never events as defined by the Centers for Medicare and Medicaid Services in pediatric patients undergoing spinal fusion procedures but does not necessarily define the predictors to avoid such events even in the highest standards of care. Future studies are needed to assess the potential modifiable risk factors in such population to minimize the occurrence of never events, which may have financial incentives [26], [27] and improvement of patient safety [28] as well as hospital quality [29]. Minimizing or avoiding such never events may be associated with benefits including decreased length of stay, hospital charges, and overall morbidity and mortality.

## Conclusions

This study provides a national estimate of the prevalence of several never events in pediatric patients who underwent spinal fusion procedures. The study further highlights certain patient characteristics that may predispose to a higher rate of occurrence of the never events. Identification and modification of risk factors may enable optimization of outcomes.
